# Human Endothelial Progenitor Cells Internalize High-Density Lipoprotein

**DOI:** 10.1371/journal.pone.0083189

**Published:** 2013-12-30

**Authors:** Kaemisa Srisen, Clemens Röhrl, Claudia Meisslitzer-Ruppitsch, Carmen Ranftler, Adolf Ellinger, Margit Pavelka, Josef Neumüller

**Affiliations:** 1 Center for Anatomy and Cell Biology, Department of Cell Biology and Ultrastructure Research, Medical University of Vienna, Vienna, Austria; 2 Institute of Medical Chemistry, Center for Pathobiochemistry and Genetics, Medical University of Vienna, Vienna, Austria; Tohoku University, Japan

## Abstract

Endothelial progenitor cells (EPCs) originate either directly from hematopoietic stem cells or from a subpopulation of monocytes. Controversial views about intracellular lipid traffic prompted us to analyze the uptake of human high density lipoprotein (HDL), and HDL-cholesterol in human monocytic EPCs. Fluorescence and electron microscopy were used to investigate distribution and intracellular trafficking of HDL and its associated cholesterol using fluorescent surrogates (bodipy-cholesterol and bodipy-cholesteryl oleate), cytochemical labels and fluorochromes including horseradish peroxidase and Alexa Fluor® 568. Uptake and intracellular transport of HDL were demonstrated after internalization periods from 0.5 to 4 hours. In case of HDL-Alexa Fluor® 568, bodipy-cholesterol and bodipy-cholesteryl oleate, a photooxidation method was carried out. HDL-specific reaction products were present in invaginations of the plasma membrane at each time of treatment within endocytic vesicles, in multivesicular bodies and at longer periods of uptake, also in lysosomes. Some HDL-positive endosomes were arranged in form of “strings of pearl”- like structures. HDL-positive multivesicular bodies exhibited intensive staining of limiting and vesicular membranes. Multivesicular bodies of HDL-Alexa Fluor® 568–treated EPCs showed multilamellar intra-vacuolar membranes. At all periods of treatment, labeled endocytic vesicles and organelles were apparent close to the cell surface and in perinuclear areas around the Golgi apparatus. No HDL-related particles could be demonstrated close to its cisterns. Electron tomographic reconstructions showed an accumulation of HDL-containing endosomes close to the trans-Golgi-network. HDL-derived bodipy-cholesterol was localized in endosomal vesicles, multivesicular bodies, lysosomes and in many of the stacked Golgi cisternae and the trans-Golgi-network Internalized HDL-derived bodipy-cholesteryl oleate was channeled into the lysosomal intraellular pathway and accumulated prominently in all parts of the Golgi apparatus and in lipid droplets. Subsequently, also the RER and mitochondria were involved. These studies demonstrated the different intracellular pathway of HDL-derived bodipy-cholesterol and HDL-derived bodipy-cholesteryl oleate by EPCs, with concomitant.

## Introduction

Endothelial Progenitor Cells (EPCs) represent a population of stem cell circulating in small fractions in human peripheral blood with the ability to proliferate, migrate and differentiate into mature endothelial cells (ECs). Asahara et al. [1] published the first detailed description of an isolation method for putative EPCs from human peripheral blood. This unique cell fraction among peripheral blood mononuclear cells (PBMNCs) derived from bone marrow was shown to be incorporated into ischemic vessels that provided tissue recovery and improvement. Since this discovery, the studies on EPCs have increasingly initiated interest of scientists working in the field of vascular biology, focused on atherosclerosis and cardiovascular diseases.

The description of putative EPCs forming clusters in vitro [1] was further extended by Hill et al. [2] who demonstrated the outgrowth of EPCs from human PBMNCs inoculated in fibronectin-coated dishes in tissue culture medium 199 supplemented with specific growth factors. After two days new clusters containing EPCs appeared which were plated again to evaluate and quantify the emergence of the colony–forming EPCs designated as CFU-Hill and characterized by a central core of “round” cells, with rather elongated “sprouting” cells at the periphery with endothelial-like morphology. These EPCs can be identified by their uptake of Dil (3,3’ dioctadecylindocarbocyanine)-labeled acetylated LDL (DIL-ac-LDL) and also by cell surface staining with Ulex europaeus agglutinin 1 (UEA-1). The CFU-Hill assay has been used to demonstrate a correlation between the circulating CFU-Hill concentration and the Framingham cardiovascular risk score in human subjects. In healthy individuals, the number of colonies correlated negatively with the Framingham cardiovascular risk score and positively with sufficient vascular function.

HDL is the smallest and densest lipoprotein that is often referred to as “the good cholesterol”. A high level of HDL-cholesterol in the blood circulation reduces the risk for heart disease while low levels increase the risk for heart disease. As long ago as 1977, the Framingham study showed that depressed levels of HDL-cholesterol were significantly and independently associated with an increased risk of coronary death [3]. Many studies showed that HDL has various direct effects on EPCs and endothelial cells (ECs). In this respect, a low plasma level of HDL is associated with decreased EPCs numbers and an impaired endothelial function [2]. HDL is able to increase the number of EPCs and to enhance their proliferation and migration [4]. It stimulates EPCs differentiation and increases EPCs contribution to angiogenesis. Additionally, HDL exerts anti-apoptotic affects on ECs and EPCs [5].

Above all, an atheroprotective effect of HDL has been demonstrated, mainly by transporting cholesterol from the peripheral vasculature and returning it to the liver via a reverse cholesterol transport pathway [6], [7]. However, there are many open questions concerning the intracellular cholesterol transport. Several pathways of intracellular cholesterol trafficking have been reported. HDL docks to a cell surface receptor, which triggers a signal, leading to the delivery of cholesterol to HDL without internalization into cellular compartments [8], [9]. Others investigators described a particular pathway of endosomal internalization and subsequent retroendocytosis of HDL particles after enrichment of cholesterol in hepatocytes, and enterocytes [9]–[11]. A study on cultured rat luteal cells showed evidence for degradation of HDL particles occurring during cholesterol uptake while the degradation products of HDL appeared as trichloroacetic acid-soluble as well as precipitable fragments [12].

Chao et al. [13] demonstrated in aortic endothelial and smooth muscle cells an HDL-mediated cholesterol efflux taking place on the surface of invaginations and within plasmalemmal vesicles located close to the cell surface. HDL was internalized into endosomes and rarely into lysosomal-like bodies [13]. Recent studies have demonstrated the intracellular location of internalized holo-HDL particles in the human hepatoma cell line (HepG2). These data showed a rapid uptake of HDL particles and a preferred location within multivesicular bodies (MVBs) and only to minor degree within lysosomes [14].

The most abundant lipoprotein within the arterial wall is HDL. It is the main acceptor for cholesterol efflux from the extrahepatic cells. In addition, HDL influences the function and survival of ECs and the repair of the endothelium of injured vessels by EPCs resulting in the prevention of endothelial dysfunction, plaque rupture, atherosclerotic lesion progression and restenosis. However, it is unknown so far how HDL is transported through ECs or EPCs.

In this study, a modified preparation protocol according to Hill et al. [2] has been applied for isolation of EPCs from buffycoats of adult donors by using density gradient centrifugation in order to characterize them and to analyze the internalization and the intracellular traffic of HDL within these cells. The aim of this study was to use EPCs as a model system since these cells represent intermediate stages of differentiation between monocytes and ECs. We used fluorescence and electron microscopical methods in combination with different labeling procedures for HDL, bodipy-cholesterol and bodipy-cholesteryl oleate including horseradish peroxidase (HRP) and the fluorochrome Alexa Fluor® 568, in order to follow their subcellular routes and to analyze morphologically the time course of human serum HDL internalization in these particular cells. We visualized the localization of fluorolabeled HDL at ultrastructural level by means of oxidation and photooxidation of diaminobenzidine (DAB), and documented the organization of the endosomal compartment, the Golgi apparatus and the TGN. We were interested to obtain more information whether or not EPCs forming a subpopulation of monocytes have the same HDL uptake and subsequent intracellular trafficking pathways as adult ECs.

## Materials and Methods

### Blood collection and cell separation

Buffycoats (60–100 ml), anticoagulated with CPD-1 (3.37 g citric acid, 26.3 g trisodium citrate, 2.22 g sodium dihydrogen phosphate, 31.8 g dextrose; and 0.257 g adenine per liter of distillated water, pH 5.6) were obtained from the blood donation center for Vienna, Lower Austria and Burgenland of the Austrian Red Cross. Samples were taken as anonymized rest material of blood donations from healthy adult voluntary donors positive for the blood group antigens AB according to the Austrian regulations for blood donation [15] after informed written consent. PBMNCs were isolated by centrifugation over a flotation medium for mononuclear cells (Ficoll-Paque, GE Healthcare Europe GmbH, Vienna, Austria) at 400g for 20 min. PBMNCs, concentrated at the interphase layer between flotation medium and citrate buffer, were collected and washed twice in EDTA-PBS by centrifugation at 125 x g for 10 min in order to remove contaminating platelets. The resuspended cells were plated on culture flasks coated with human fibronectin (FN; Sigma-Aldrich, Vienna, Austria) for 12 hours and cultivated in medium 199 (Sigma-Aldrich, Vienna, Austria) supplemented with 20% fetal calf serum (FCS), 200 mM L-glutamine, antibiotics (100 IU/ml penicillin, 100 µg/ml streptomycin; all from PAA Laboratories, Pasching, Austria) and 50 ng/ml human recombinant vascular endothelial growth factor (rHu VEGF_165_, PromoKine, Heidelberg, Germany) at 37°C in a 95% humidified air and 5% CO_2_ tension. One day after seeding, non-adherent PBMNCs were transferred to another FN-coated flask to remove circulating (mature) endothelial cells (CECs) adhering on the growth surface of the 1^st^ flask. Medium changes were performed twice a week. The cells were grown until pre-confluence (approximate 10 days) before starting the experiment. The cells were removed from the flasks using trypsin-EDTA (PAA Laboratories, Pasching, Austria) seeded on FN-coated round glass coverslips and plated at the bottom of 8-well plates (Merck, Darmstadt, Germany), maintained in medium 199 supplemented with 20% FCS, 2 mM L-glutamine and antibiotics. 1 day before starting the experiment, cells were washed twice with phosphate buffered saline (PBS) and fed with medium 199 containing 20% calf lipoprotein deficient serum (cLPDS).

### EPCs characterization

EPCs were characterized by lectin binding. Cells grown on FN-coated round glass coverslips were washed with Ca^++^ and Mg^++^-free PBS and fixed at 4°C in CellFix (Becton Dickinson, Vienna, Austria) for 10 min, washed again twice with PBS, preincubated with PBS containing 5% FCS in order to block unspecific binding sites. Labeling with biotinylated UEA-1 (10 mg/ml; Sigma-Aldrich,Vienna, Austria) was carried out at concentration of 20 µg/ml in 500 µl of PBS containing 5% FCS for 1 hour at 4°C. As a second step reagent, phycoerythrin (PE)-conjugated streptavidin (Becton Dickinson, Vienna, Austria) was used.

In order to see whether or not EPCs express the scavenger receptor B1 (SR-B1) at their surface, a double labeling was performed using a purified monoclonal mouse IgG_1_ antibody against the SR-B1 receptor (analogues: CL-A1, CD36 and LIMPII; clone 25/CLA-1; 250µg/ml) Becton Dickinson, Vienna, Austria) and a polyclonal goat-anti-mouse antibody, conjugated with Alexa Fluor® 488 (LifeTech Austria, Vienna, Austria) as 2^nd^ step reagent as well as with rhodamin-conjugated UEA-1 (10 mg/ml; Vector Laboratories, LTD, Peterborough, UK).

Intracellular von Willebrand factor (vWF) was demonstrated using a FITC-conjugated polyclonal sheep anti-human antibody (Serotec, Oxford, UK). After cell fixation at 4°C in CellFix, a permeabilization step with 100% methanol was carried out for 15 min at –20°C, followed by washing and labelling with the vWF antibody in PBS containing 5% FCS for 20 min at 4°C. In all cases, fixed samples were washed extensively and the coverslips were mounted with Fluoroprep (Biomerieux, France) on microscopy slides, and investigated using an inverted fluorescence microscope (Nikon, Eclipse TE-300, Nikon Coop., Tokyo, Japan) equipped with a HBO lamp and conventional filter packs for blue and green light excitation. For image documentation, the microscope was connected with a Nikon Coolpix 5000 digital camera DS-5M.

### Preparation of HDL and labelling procedures

Plasma was collected from healthy donors and HDL was separated at density of 1.21 g/ml using sequential flotation ultracentrifugation [16]. The density of the plasma fraction was adjusted with potassium bromide (KBr). HDL was then dialyzed extensively against 0.9% NaCl and 0.1% EDTA, pH 7.4 at 4°C for 24 hours.

HDL-HRP conjugates were prepared using the peroxidase labeling kit (Roche, Basel, Switzerland) according to the manufacturer’s instruction. The labeling procedure for HDL- Alexa Fluor® 568, HDL-bodipy-cholesterol, HDL-bodipy-cholesteryl oleate has been previously described [16], [17], [18]. Bodipy –cholesterol and Bodipy-cholesteryl oleate were kind gifts of Dr. Robert Bittman (Queens College, The City University of New York).

### HDL-HRP binding and uptake

Cells were incubated with labeled HDL (HRP-HDL 50 µg/ml) diluted in medium 199 containing 2 mg/ml of fatty acid-free bovine serum albumin (faf-BSA) at 37°C for 30 min, 1, 3 and 4 hours. The use of fatty acid-free BSA is very important HDL uptake studies since fatty acid-loaded BSA leads to a partial or complete saturation of the corresponding receptors. After the uptake experiments, cells were washed with PBS and fixed with 2.5% glutaraldehyde in PBS, pH 7.4 for 30 min. Cell were pre-incubated in 1 mg/ml 3,3′ diaminobenzidine tetrahydrochloride (DAB; Sigma-Aldrich, Vienna, Austria) in 0.1 M Tris (2-Amino-2-hydroxymethyl-propane-1,3-diol) buffer, pH 7.4, at room temperature for 15 min. After discarding the preincubation solution, the cells were incubated again for 30 min in the same solution, additionally containing 1% H_2_O_2_ in order to initiate the peroxidase reaction. The incubated samples were postfixed for 15 min at 4°C in 1% veronal acetate-buffered OsO_4,_ pH 7.2 [18]. After washing, cells were processed for routine electron microscopy.

### HDL-Alexa Fluor® 568, HDL-bodipy-cholesterol and HDL-bodipy-cholesteryl oleate binding and uptake demonstrated by DAB photooxidation

Cells were incubated with Alexa Fluor® 568-labeled HDL (50 µg/ml), HDL bodipy-cholesterol (50 µg/ml) or HDL bodipy-cholesteryl oleate (50 µg/ml) diluted in medium 199 containing 2 mg/ml faf-BSA at 37°C for 30 min, 1, 3 and 4 hours. After the uptake of these substances, cells were subjected to photooxidation. This technique is used to convert fluorescent signals in electron-dense precipitates allowing their localization in the electron microscope as recently described [17]. Briefly, cells were fixed with 4% formaldehyde and 0.5% glutaraldehyde in PBS at 4°C for 45 min. After washing in PBS, cells were preincubated in a 0.1% solution in 0.05 M Tris-HCL buffer, pH 7.4 in an ice bath. After changing this solution, illumination was performed for 20 min with a 100 W HBO lamp at an inverted microscope (Nikon Eclipse TE300) using a 40x objective. After rinsing cells in distilled water twice for 5 min, they were postfixed with 1% osmium-ferrocyanide (1:1 mixture of 2% aqueous OsO_4_ and 3% potassium ferrocyanide (K_4_Fe(CN)_6_, Merck, Darmstadt, Germany) at room temperature for 15 min followed by an incubation for 4 hours at 4°C in 1% veronal acetate-buffered OsO_4_ pH.7.2 followed by processing for routine electron microscopy.

### Transmission electron microscopy (TEM)

The labeled cells were dehydrated in graded series of ethanol (50, 70, 90, 96, and two times 100%) and embedded in Epon (Serva, Heidelberg, Germany). Ultrathin sections (80–100 nm) were cut using an UltraCut-UCT ultramicrotome (Leica Inc., Vienna, Austria), transferred to copper grids and viewed either unstained or stained with 1% uranyl acetate and 5% lead citrate (Merck, Darmstadt, Germany) using an EM-900 TEM (Carl Zeiss, Oberkochen, Germany) at an acceleration voltage of 50 kV. Digital images were recorded using a wide-angel dual speed CCD camera (Albert Tröndle, Dünzelbach, Moorenweis, Germany).

### Electron tomography (ET)

ET was performed using a Tecnai 20 TEM (FEI, Eindhoven, The Netherlands) at 200 kV by acquisition of tilt series at a tilt range of ± 65° with an increment of 1° using a 1k slow scan CCD camera (Gatan, Munich, Germany). The digital images were stored into stack files (*.mrc). The acquisition was triggered by the Explore 3D software (FEI) which allows compensating dislocations of the region of interest during tilting. Alignment by sequential cross-correlation and reconstruction of tilt series by the weighted back projection (WBC) method were carried out using the Inspect 3D software (FEI) resulting in volumes consisting of virtual slices from which 3D models were created using the Amira 4.1 software (Mercury Computer Systems, Merignae, Cedex, France).

## Results

### Morphological characterization of EPCs

**Figure 1:**
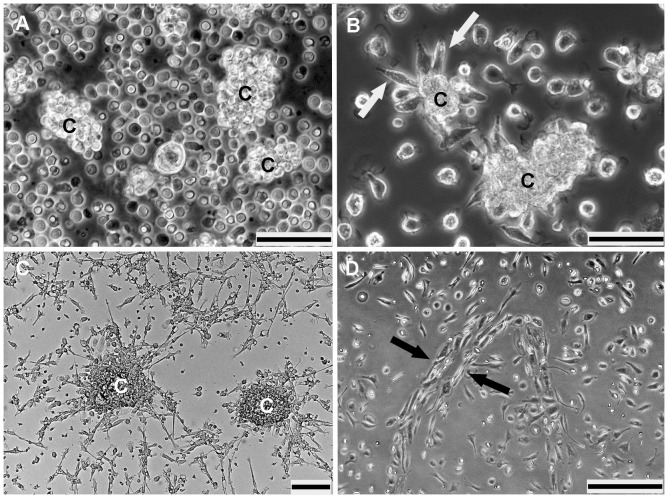
Differentiation of PBMNCs derived EPCs after isolation and seeding onto FN-coated flasks with medium 199 shown in phase contrast. **A**) PBMNCs, 30 min after seeding form many large and small clusters (C). **B**) They are able to differentiate into spindle cells 48 hours after culture (white arrows). **C**) Most of the attached cells become spindle shape on day 5 in culture; cell clusters (C). **D**) Spindle shaped morphology form tubular like structure (black arrows) after 7 days in culture. (Scale bar: A and B = 50 µm; C and D = 100 µm).

After isolation, the inoculated mononuclear cells (PBMNCss) appeared as round cells forming non-adherent clusters which later became the centers of high proliferation (Figure 1A). Non-adherent cells were separated from slightly adherent cells containing mature ECs (circulating ECs which were present in the peripheral blood of the donor) by transfer into a new FN-coated flask. One day after the transfer, cell clusters adhered to the growing support of this flask. After 2-3 days of culture, these colonies gave rise to spindle-shaped cells (Figure 1B) representing the EPCs as previously described [1]. These cells appeared either as single cells or as clusters comprising round cells centrally and sprouts of spindle shaped cells at the periphery (Figure 1C). Single or double rows of aligned cells resembling vessel-like tubular structures occurred after 7 days of culture (Figure 1D). 

### Identification of EPCs growing out from PBMNCss clusters from a blood group antigen AB positive donor using cytochemical methods at light and electron microscopical level

**Figure 2:**
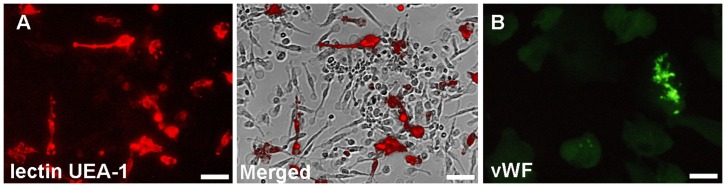
Phenotypic characterization of EPCs after 10 days in culture. **A**) **A part of t**he attached spindle-like cells are positive for UEA-1 shown also as an overlay from a fluorescence and a phase contrast image. **B**) EPCs express the endothelial markers vWF. (Scale bars in A and B = 50 µm).

EPCs characterization by cell surface staining with the biotinylated lectin UEA-1 in combination with streptavidin-PE was demonstrated by their discrimination from other mononuclear cells using overlays of fluorescence and phase contrast images. Approximately, one third of the adherent cells were UEA-1 positive (Figure 2A). Using a FITC-labeled polyclonal antibody, a small portion of EPCs showed fluorescent patch-like vWF positive structures resembling Weibel-Palade bodies (Figure 2B) but also a homogenous intracellular vWF-related fluorescence.

### Determination of cell surface expression of the SR-B1 receptor

**Figure 3:**
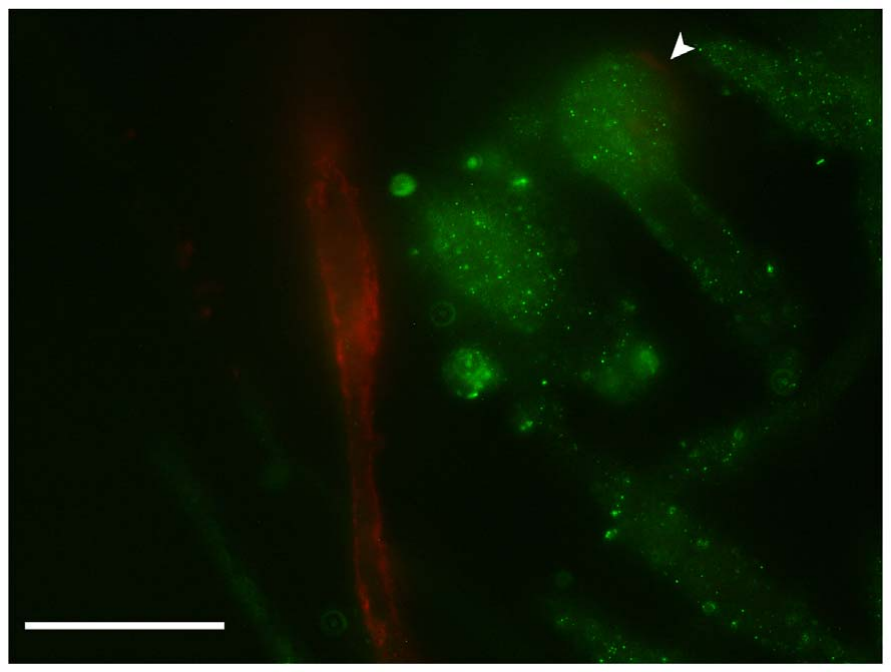
Labeling of cells outgrowing from monocytic clusters with UEA-1, and anti-SR-B1 antibody and goat-anti-mouse antibody conjugated with Alexa Fluor® 488 as 2^nd^ step reagent. A distinct green fluorescence is visible in the majority of outgrowing cells. Note that UEA-1 binding EPCs are negative for the SR-B1 receptor with exception of 1 cell which is weakly positive for UEA-1 (arrowhead). (Scale bar  = 20 µm).

The majority of spindle-like cells growing out from monocytic clusters exhibited a positive reaction with the SR-B1 antibody (green fluorescence in Fig. 3). Contrastingly, EPCs characterized by the binding of UEA-1 (red fluorescence in Fig. 3) were negative for the SR-B1 receptor with exception of few SR-B1 positive cells, binding UEA-1 only weakly (arrowhead).

### HDL-HRP binding and uptake

**Figure 4:**
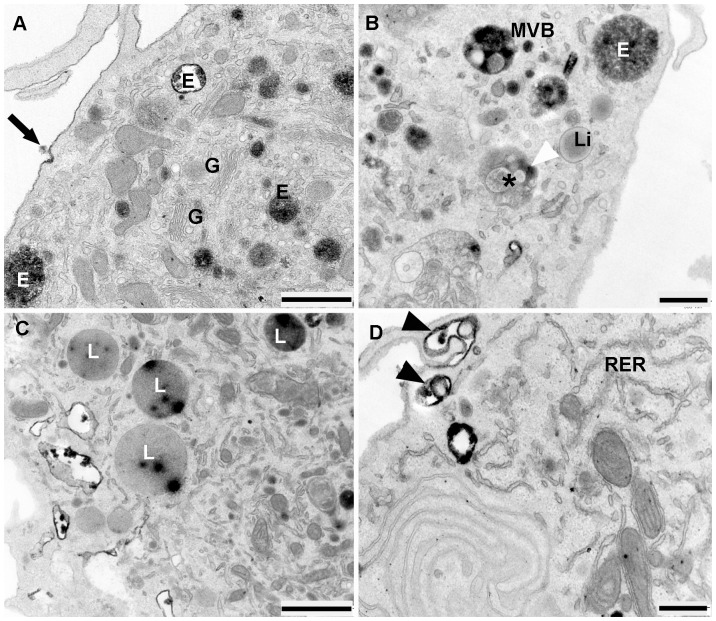
Ultrastructural appearance of EPCs after 30 minute of HDL-HRP internalization. **A**) HDL-HRP particles can be seen at the cell surface (black arrow), the various sizes of HDL-HRP positive endosomal vesicles (E) are present beneath the cell membrane and close to the Golgi apparatus (G). **B**) HDL-positive and -negative MVBs (MVBs, asterisk) are visible. The white arrowhead indicates HDL-negative MVBs representing an early stage of lipid internalization; lipid droplets (Li) appear unstained. **C**) Lysosomes (L) shows intraluminal HDL-HRP particles. **D**) The RER is HDL-HRP negative while two invaginations of the plasma membrane (black arrowheads) show a strong reaction. (Scale bar: A, C = 1 µm; B, D = 0.5 µm).

HDL-HRP internalization studies provided intense reactions at invagination sites of the plasma membrane at each time of treatment. With increasing times of treatment, HDL-HRP was internalized and accumulated in multiple intracellular compartments. After incubation time for 30 min, the HDL-HRP particles were not only distributed on the cell surface at sites of plasma membrane invagination but also inside of cytoplasmic vesicles of varying sizes, representing early endosomes displaying an intensive DAB reaction (Figure 4A-D). HDL-positive and-negative MVBs could also be observed at the cell periphery (Figure 4B). The lumina of those positive MVBs were filled with reaction product but most of the intraluminal vesicles remained unstained. Internalized HDL-particles were also found in lysosomes, which were characterized by a homogenous luminal electron-dense granularity (Figure 4C). In contrast, lipid droplets, the RER as well as the Golgi apparatus remained unstained (Figure 4A, B, D).

**Figure 5:**
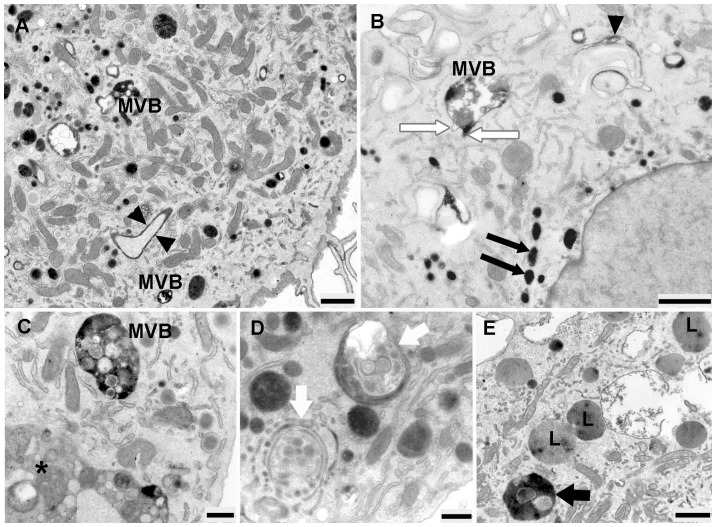
Micrographs of EPCs at 1 hour of incubation with HDL-HRP. **A**) Numerous positive endosomal vesicles of varying sizes are found throughout cytoplasm, membrane invagination are indicated by arrowheads, positive MVBs are present. **B**) Positive MVBs with irregular shape and tubular appendices (white thin arrows), an endosome alignment (black thin arrows) as well as membrane invagination (black arrowhead) are demonstrated. **C**) Showing positively and negatively stained MVBs (asterisk). **D**) The large structures represent autophagosomes (white thick arrows) containing endosomes, are enclosed by a multilamellar membrane. **E**) The HDL-HRP particles are apparent in lysosomes (L, black thick arrow) (Scale bar: A, B, E = 1 µm; C, D = 0.5 µm).

After 1 hour of incubation, HDL-HRP particles were detected in variably sized endosomal vesicles throughout the cytoplasm (Figure 5A). Some of them were arranged as endosome alignments (Figure 5B). The positively and negatively stained MVBs were irregularly shaped (Figure 5B, C), some of them possessing tubular membranous extensions. Autophagosomes, enveloped by multilamellar membranes frequently contained endosomes and/or lysosomes. (Figure 5D). In addition, HRP-DAB staining was located in lysosomes (Figure 5E).

**Figure 6:**
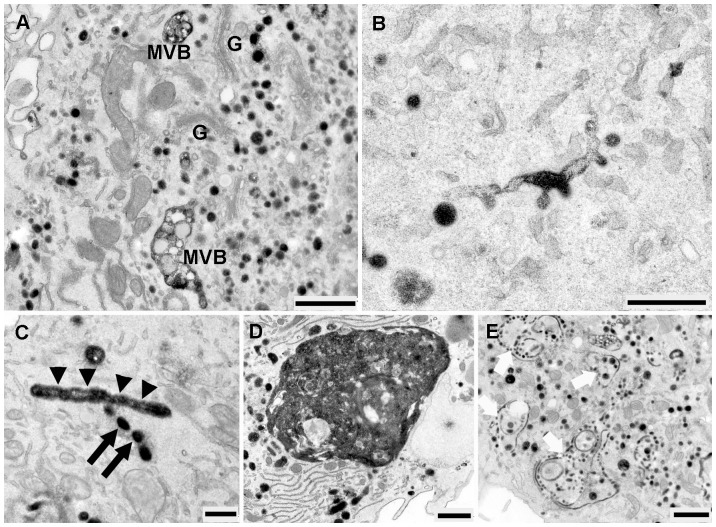
Appearance of EPCs after 3 hour of HDL-HRP internalization. **A**) The population of positively stained endosomal vesicles increase and could be observed throughout the cytoplasm. Positive MVBs (MVB) in various sizes and shapes with tubular membranous extensions as well as prominent Golgi stacks (G) are visible. **B**) This figure shows the appearance of a tubular endosome. **C**) Arrows point to an endosome alignment forming “strings of pearl-like structures” (arrowheads). **D**) A large secondary lysosome shows intraluminal HDL-HRP particles. **E**) Numerous autophagosomes (white thick arrows) with included endosomal vesicles are noted. (Scale bar: A, D, E = 1 µm; B, C = 0.5 µm).

At 3 hours of incubation, positively stained organelles were distributed throughout the cells, concentrated at perinuclear areas in close association to the Golgi apparatus (Figure 6A). The population of positively stained organelles as well as the MVBs with irregular shape and tubular membranous extensions increased with incubation time. Also tubular endosomes could be seen (Figure 6B). Interestingly, HDL containing endocytic compartments, arranged as endosomal alignments appeared partially fused, forming “strings of pearl-like structures” (Figure 6C). Furthermore, large secondary lysosomes, approximately 2 µm in diameter (Figure 6D) as well as an accumulation of autophagosomes consisting of multimembranous structures and containing endosomal vesicles could be demonstrated (Figure 6E).

**Figure 7:**
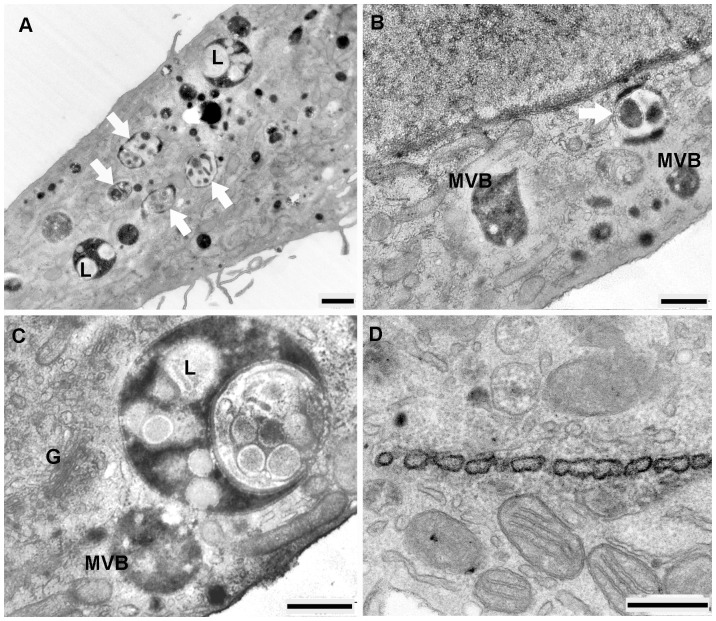
Ultrastructural appearance of EPCs after 4 hour of incubation with HDL-HRP. **A**) Autophagosomes (white thick arrows) with engulfed endosomal vesicles are noted. **B**) Positive MVBs with the characteristic tubular membranous extensions are visible. **C**) High magnification of figure 6a, shows a large spherical secondary lysosomes (L) close to Golgi apparatus (G). **D**) Demonstration of “strings of pearls-like structures”. (Scale bar: A = 1 µm; B, C, D = 0.5 µm).

At 4 hour of incubation, the number of stained organelles as well as of MVBs decreased in comparison to other time points of the experiment (Figure 7A, B). Lysosomes showed intraluminal HDL-HRP particles, located close to Golgi apparatus (Figure 7C). They were spherically shaped with approximately 500 nm in diameter and bordered by a double membrane. Also multimembraneous autophagosomes, contained endosomal vesicles could be detected (Figure 7A, B). In addition, also the characteristic “strings of pearl-like structures” could be observed (Figure 7D).

Concerning the different incubation times with HDL-HRP, the number of stained organelles increased with longer incubation periods, the labeled endocytic vesicles and lysosomal structures appeared in regions close to cell surfaces and were concentrated in perinuclear areas in close association to Golgi apparatus. However, HDL-HRP could never be demonstrated in the Golgi apparatus. In control experiments, using only peroxidase-containing medium without HDL no DAB reactions occurred.

### Electron tomography (ET)

**Figure 8:**
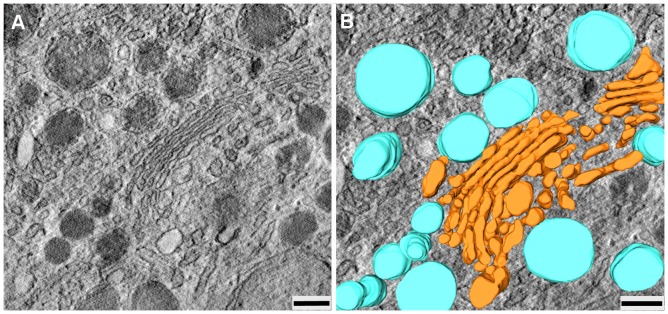
ET indicates HDL-containing endosomes. **A**) shows a virtual slice, **B**) a corresponding model with endosomes (bright blue color) are accumulated in close proximity to the Golgi apparatus (orange color). (Scale bar: A, B = 200 µm).

The analysis of ET tilt series and 3D reconstruction of HDL-positive compartments after 3 hour of HDL internalization revealed a special arrangement of differently shaped HDL-HRP positive endosomes accumulating in perinuclear areas around the Golgi apparatus (Figure 8).

### HDL-Alexa Fluor® 568 binding and uptake

To further substantiate that EPCs internalize HDL, we used a different approach to follow HDL endocytosis: HDL-Alexa Fluor® 568 internalization routes were visualized by means of the DAB photooxidation technique which has been previously applied for transport of Bodipy-ceramide into the TGN [19], [20] or to monitor trafficking of lipids derived from lipoprotein particles [17]. This method allows conventional fluorescence microscopy as well as conversion of fluorescence signals into an electron dense DAB precipitate without preceding membrane permeabilization as it is necessary by the use of HRP-labeled antibodies.

**Figure 9:**
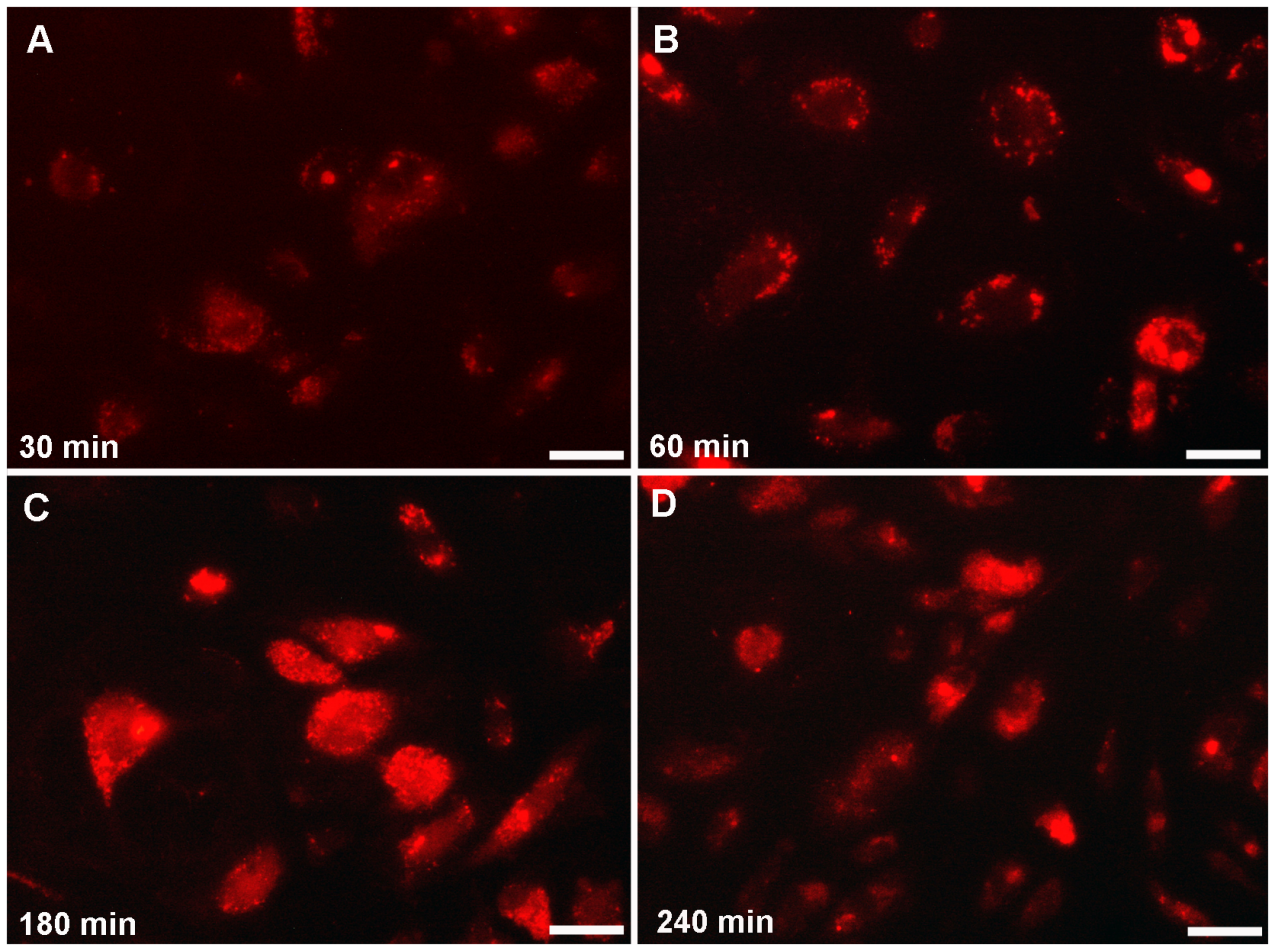
Time course of HDL-Alexa Fluor® 568 internalization after interval from 30 to 240 min shows the fluorescence-marked structures in bright signals increasing in size and number of intracellular endocytic compartments. (Scale bar: A, B, C, D = 50 µm)

The result of HDL-Alexa Fluor® 568 internalization at time periods from 30 to 240 min demonstrated by fluorescence microscopy, showed an increase over time in size and number of bright staining fluorescence signals of intracellular endocytic compartments distributed throughout the cell (Figure 9 A-D). However, after 240 min of internalization, the fluorescent organelles decreased (Fig. 9D). These findings are in line with the ultrastructural result shown below.

**Figure 10:**
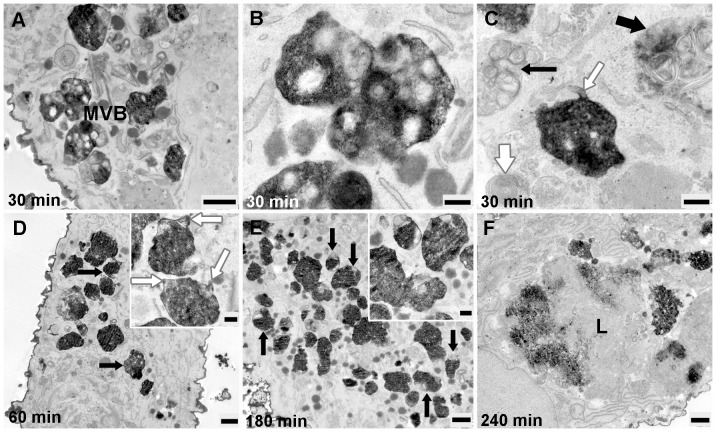
The internalization of EPCs with HDL-Alexa Fluor® 568 between 30 and 240 min. **A**) Positively stained organelles and MVBs are at cell periphery. **B**) High-power micrograph of positive MVBs demonstrating the densely stained limiting and multilamellar vesicle membranes. **C**) A HDL-Alexa Fluor® 568 positive MVBs shows tubular membranous extension (white thin arrow); unstained MVBs (black thin arrow), an autophagy vacuole with whorls of membranous material surrounding (white thick arrow) and autolysosome (black thick arrow) are observed. **D**) Black thin arrows indicate densely stained MVBs with the presence of tubular membranous appendices (white thin arrows in inset). **E**) A large number of intensely stained organelles as well as MVBs (black thin arrows) are accumulated in the deeper cell interior, the intraluminal vesicles of MVBs could rarely be found (inset). Numerous caveolae are present. **F)** The reaction products could be observed in lysosome (L)**.** (Scale bar: A, D, E, F = 1 µm; B, C, D inset  = 0.25 µm; E inset  = 0.5 µm)

The ultrastructural appearance of HDL-Alexa Fluor® 568 stained compartment in the period between 30 and 240 min of internalization, exhibited a decoration of plasma membranes with layers of electron-dense reaction products. Multiple small cytoplasmic vesicles as well as round to oval shaped MVBs were strongly positive for HDL-Alexa Fluor® 568. Typical HDL-Alexa Fluor® 568 -positive MVBs were characterized by a large number of equally stained intra-vacuolar vesicles as well as an intensive staining of the limiting and vesicular membranes. Most of these organelles showed multilamellar membranes that became more distinct as they were larger and were more loosely arranged (Figure 10A, B). In most cases, MVBs were strongly stained, their intraluminal vesicles were overlaid by positively stained reaction product resulting in a difficulty to detect their internal luminal vesicles (Figure 10C-E). Nevertheless, they could be identified as MVBs by the presence of tubular membranous extensions (Figure 10C, D). HDL-stained compartments as well as MVBs were frequently concentrated in close spatial relationship to the unstained Golgi apparatus. Unstained MVBs with multilamellar limiting and vesicular membranes were visible (Figure 10C). Individual autophagosomes appeared as whorls of membranous material, some of them could be observed together with lysosome to form autolysosomes (Figure 10C). Some reaction products could also be detected in lysosomes (Figure 10F).

The number and the intensity of HDL-stained compartments as well as MVBs increased with the time of uptake. The highest number of stained organelles was detected at 180 min, while at 240 min their number decreased in comparison to other incubation times.

### HDL-derived Bodipy-Cholesterol uptake and binding

**Figure 11:**
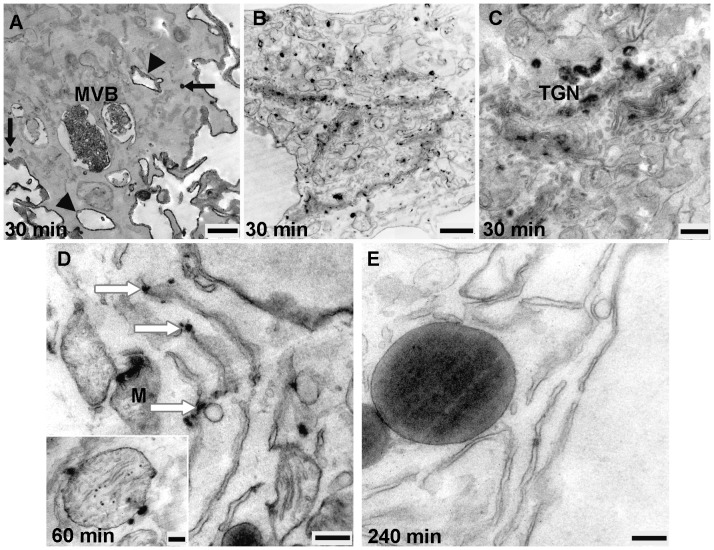
Micrographs of EPCs incubated with HDL labeld bodipy-cholesterol between 30 and 240 min. **A**) Small cytoplasmic vesicles are stained (black thin arrows), the large 2 positive MVBs exhibit tightly-packed intraluminal microvesicles with reaction products. **B**), **C**) Reaction products are widely dispersed in all parts of the Golgi apparatus and the TGN. **D**) RER (white arrows) and mitochondria (M and in inset) are weakly stained. **E**) Demonstration of close association between unlabeled lipid droplets and RER. (Scale bar: A = 0.5 µm; B = 1 µm; C, D, D inset, E = 0.25 µm).

HDL transports cholesterol as both free and esterified cholesterol. Previously, we and others have characterized Bodipy-labeled cholesterol surrogates as suitable probes to analyze the cellular trafficking of these lipids [17], [21]. The ultrastructural analysis of EPCs incubated with HDL labeled with bodipy-cholesterol showed a different uptake pattern compared to the experiments with HDL-HRP and HDL-Alexa Fluor® 568. After 30 min of incubation, small cytoplasmic vesicles as well as large positive MVBs containing intraluminal microvesicles, tightly-packed with reaction products could be demonstrated (Fig. 11A). Furthermore, a high amount of reaction product was found to be localized within all parts of the Golgi apparatus including the TGN (Figure 11B 11C) whereas mitochondria and RER were only weakly stained (Figure 11D). In the period between 60 and 240 min, positive and negative MVBs of varying sizes were visible. The internalized-HDL-derived bodipy-cholesterol was spread within many of the stacked Golgi cisterns and the TGN (Figure 11C), whereas the lipid droplets were unstained (Figure 11E). Lysosomes and large structures of autophagy were also present.

### HDL-derived Bodipy-Cholesteryl Oleate uptake and binding

**Figure 12:**
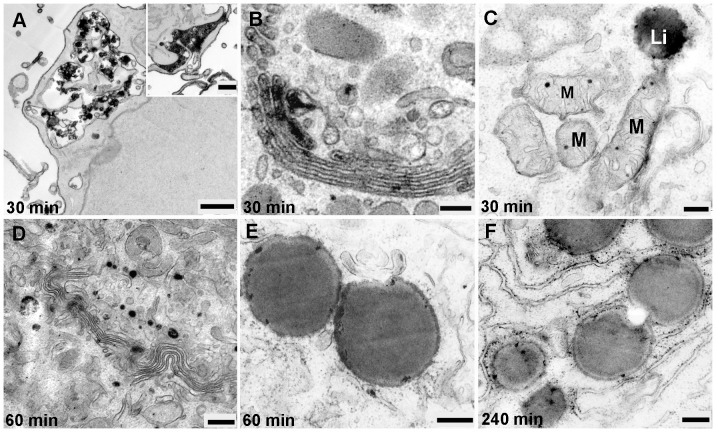
Ultrastructural detection of the internalization of EPCs with HDL-bodipy-cholesteryl oleate between 30 and 240 min. **A**) MVBs at the cell periphery are filled with numerous tightly packed positively stained microvesicles; the membrane invagination are full filled with the reaction products also present on the other side of cell (inset). **B**), **D**) The TGN and stacked Golgi cisternae are positively stained. **C**) Mitochondria (M) and lipid droplet (Li) are labeled. **E**), **F**) A close association between labeled lipid droplets and RER could be demonstrated. (Scale bar: A = 1 µm; A inset = 0.5 µm; B, C, D, E, F = 0.25 µm).

After 30 min of incubation, TGN and stacked Golgi cisternae were positive for DAB reaction products (Figure 12A, B and D), mitochondria were weakly stained and the lipid droplets started to accumulate Bodipy-cholesteryl oleate (Figure 12C). In the period between 60 to 240 min, oval positive MVBs were visible both at the cell periphery and at the perinuclear region. The lipid droplets were stronger labeled than was seen after 30 min and reaction products were demonstrated at their limiting membranes. Labeled RER was also frequently present in close proximity to the labeled lipid droplets (Figure 12E, F). The highest numbers of stained organelles were detected after 180 min of incubation, located in close vicinity of the stained Golgi apparatus. The lysosomes could be demonstrated close to autophagosomes. They were spherically shaped and exhibited positive tiny granules located in their lumen.

## Discussion

Numerous studies have demonstrated that HDL influences function and survival of ECs and regenerating EPCs. The effective repair of the endothelium results in the protection against the development of atherosclerosis and coronary artery disease. HDL and their main protein constituent, the apolipoprotein A-I (apo A-I), must leave the plasma compartment and cross the endothelial barrier to exert this atheroprotective effect within the arterial wall. Concerning the lipoprotein transport pathway, there are only few experimental data supporting the LDL transcytotic process [22], [23]. Other authors favor the view of LDL passive filtration [24], [25] while some reports focus on the transendothelial HDL transport [26], [27]. In the present study, EPCs have been used in order to investigate the transendothelial vesicular transport and the intracellular localization of internalized HDL particles. We were interested whether or not monocytes-derived EPCs follow the same HDL transport pathway as adult ECs.

**Figure 13:**
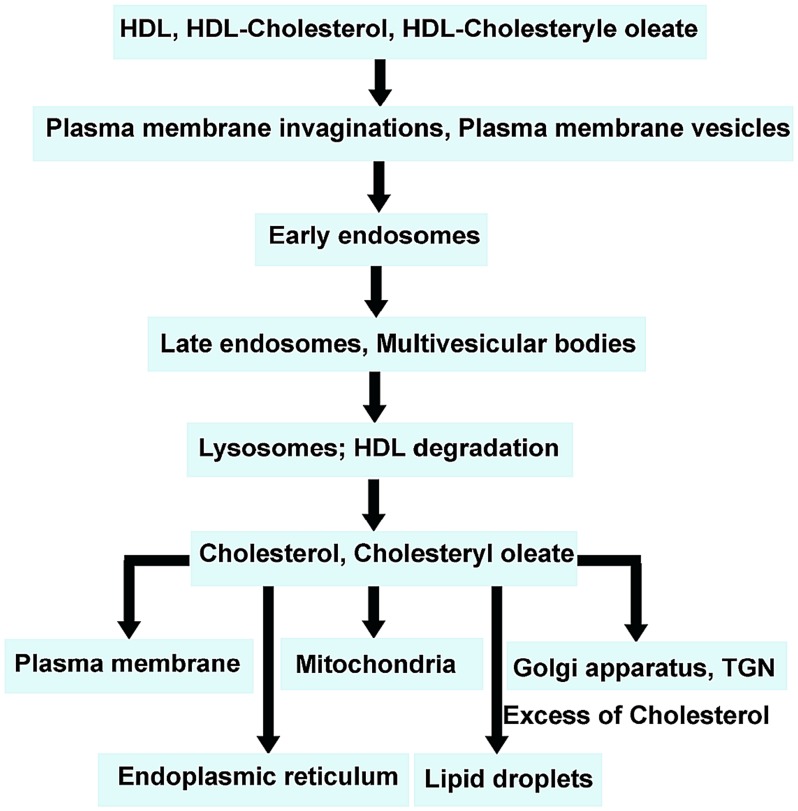
Flow chart summarizing the intracellular traffic routes of HDL, HDL cholesterol and HDL cholesteryl oleate.

We investigated the subcellular routes of HDL ultrastructurally by means of cytochemical methods using HRP-labeled HDL or photooxidation, both resulting in an electron-dense DAB precipitate. Time-kinetic experiments revealed that HDL particles were present both on the plasma membrane as well as intracellularly. We could demonstrate that internalized HDL-HRP and HDL-Alexa Fluor® 568 were accumulated in the lumen of early endosomes at the cell periphery and transported to late endosomes in the perinuclear region. Finally, late endosomes merged into lysosomes containing DAB reaction product in their lumen already at 30 minute of incubation and increasingly with longer incubation periods. For the sake of clarity, our results are summarized in a flow chart (Figure 13).

It is generally accepted that internalization and degradation of HDL is low compared to LDL [28]. However, several investigators described uptake and lysosomal degradation of HDL apolipoproteins in specialized cell types such as in rat luteal cells [12], epithelial cells of the rat visceral yolk sac trophoblasts [29], [30] and a cell line adapted to grow under low cholesterol conditions [31]. Our findings on the transport of HDL to lysosomes could not be substantiated by biochemical degradation assays. This is because successful quantification of HDL degradation with these assay requires a rather large amount of cellular material, which cannot be obtained from human sources.

Similar experimental data could be obtained investigating cultured bovine aortic endothelial cells (BAECs) and human umbilical vein endothelial cells (HUVEC) underlining the evidence of binding, internalization and transport of apo A-I-gold particles to various intracellular vesicular compartments [27]. However, they appeared rarely localized in lysosomes. Similar investigations described the evidence of plasmalemmal invaginations and vesicles during HDL-colloidal gold internalization in aortic endothelial cells [13]. It has been demonstrated that the protective action of HDL on endothelium is mediated by the ATP-binding cassette transporter A1 (ABCA1) upregulating the adenosine monophosphate activated protein kinase (AMPK) and inducing the efflux of 7-ketocholesterol and 7-ketocholesterol induced reactive oxygen species expression [32]. An additional cholesterol transporter is the ATP-binding cassette transporter G1 (ABCG1) which is localized not only at the plasma membrane but also at endosomal membranes [33], presumably also the Ecto-F1-ATPase [34] as well as the scavenger receptor B-I (SR-BI). It is generally accepted that the SR-BI is primarily the mediator for the selective transfer of lipids from lipoproteins to cells. This receptor is highly expressed in hepatocytes and steroidogenic cells, but also in endothelial cells. [35], [36]. The expression of SR-BI in cultured endothelial and smooth muscle cells of the rat aorta after exposure to HDL is specifically localized on the cell surface and favors the view that HDL may use this receptor to promote cholesterol efflux from the atheromatous arterial wall [37]. Others investigators demonstrated that bovine arterial ECs (BAECs) transcytose HDL by mechanisms that involve either the SR-BI receptor or the ABCG1 receptor [38]. In cultured cells, SR-BI appears to be concentrated in plasma membrane microdomains called caveolae [39]. These structures are known primarily for their ability to transport molecules across endothelial cells. In our experiments, plasma membrane invaginations resembling caveolae decorating the wall of newly formed vesicles were also detected which internalized HDL during pinocytosis. Binding and pinocytosis appeared randomly distributed over the whole cell surface. We suggest that an increased concentration of HDL leads to a caveolar expansion by formation of membrane invaginations and vesicles. However, early EPCs derived from monocytes did not react with the SR-B1 antibody we used. We suggest that the SR-B1 receptor is not or incompletely expressed in EPCs at this stage of endothelial differentiation. This is astonishing since the surrounding spindle-like cells appearing in the cultures of outgrowing monocytic clusters were clearly positive for this receptor. Nevertheless, other receptors such as the HDL receptor Ecto-F1-ATPase were found in human endothelial progenitor cells obtained from peripheral blood of young healthy subjects, separated by density gradient centrifugation and cultured on fibronectin-coated plates [40].

Introducing the DAB photooxidation technique, we were able to analyze membranous compartments involved in cholesterol trafficking. We could demonstrate that after uptake, HDL-derived bodipy-cholesterol as well as bodipy-cholesteryl oleate were present in the endocytic compartments and directed into the intracellular lysosomal pathway. The HDL-derived bodipy-cholesterol was widely dispersed within many of the stacked Golgi cisternae and the TGN, as well as localized in mitochondria and rarely in the ER. While HDL-derived bodipy-cholesterol was trafficking only through the lysosomal pathway, HDL-derived bodipy-cholesteryl oleate accumulated prominently in all parts of the Golgi apparatus and in lipid droplets and subsequently also in the RER and mitochondria.

Cholesterol is the most abundant sterol in mammalian cells and an essential component of the cell membrane, it is crucial for cell viability and function. Cholesterol homeostasis is maintained by the endocytic as well as by the biosynthetic pathway. Cholesterol traffic involves transport within lipoproteins (mainly low density lipoprotein) from early to late endosomes as well as from late endosomes to lysosomes [41], [42]. In accordance with a study in rat hepatoma cells [43] we could demonstrate in EPCs that cholesterol is transported from lysosomes to the Golgi apparatus from which an HDL or HDL apo-lipoprotein-mediated cholesterol transport to the plasma membrane starts. A delivery of cholesterol into the extracellular space takes place either via the endomembrane system or shuttled via a Golgi-bypass route mediated by cytosolic lipid transfer proteins (LTPs) which requires the presence of an appropriate extracellular cholesterol acceptor [44]. Nevertheless, it has been shown, that fibroblasts as well as macrophages can esterify and store excess cholesterol to a certain amount without metabolizing or excreting them [45], [46].

It could be demonstrated in rat granulosa cells that HDL-bodipy-cholesteryl oleate internalization is a rapid process followed by an accumulation of the lipid in the TGN and in lipid droplets [47]. These observations are consistent with our results. At this point, we suggest that both bodipy-cholesterol and bodipy-cholesteryl oleate may be simultaneously segregated from HDL particles in lysosomes and delivered to other membranes such as the plasma membrane and mitochondria. From lysosomes, cholesterol can become either esterified or transferred to the Golgi complex and the TGN; forming a pool of disposable free cholesterol. In addition to intracellular cholesterol exchange, also selective lipid uptake on the plasma membrane mediated by SR-BI accounts for the different trafficking route of HDL lipids and apolipoproteins.

We found that cholesterol was rarely localized in the ER. This is in line with the fact that cholesterol turnover in the ER is quick and occurs without accumulation of cholesterol in the ER. According to the intracellular staining pattern we suggest that cholesteryl oleate is increasingly delivered to the ER and subsequently stored inside of lipid droplets and that the excess of cholesterol becomes re-esterified. In this respect, our findings in EPCs demonstrate the close association evidence between the stained ER and the labeled lipid droplets.

Our results on EPCs, incubated with HDL-HRP and HDL-Alexa Fluor® 568, showed frequently positive reaction products in endocytic vesicles and organelles localized in close proximity to the Golgi apparatus but not in the Golgi apparatus itself. Contrastingly, when the cells were incubated with HDL-bodipy-cholesterol and bodipy-cholesteryl oleate, the Golgi apparatus and the TGN were involved and also the labeled organelles were found close to them. These findings indicate that HDL itself cannot be directed to the Golgi apparatus and the TGN. The close association of HDL-positive organelles with the Golgi apparatus might be responsible for an efficient delivery of cholesterol from HDL containing organelles to Golgi apparatus.

However, several studies support the view of a non-lysosomal intracellular pathway and subsequent retroendocytosis of the HDL particles after enrichment with cholesterol in hepatocytes and enterocytes [10], [11]. Another investigation has also demonstrated that HepG2 cells were not only able to bind, internalize and degrade HDL but also to resecrete the lipoprotein by the process of retroendocytosis [48]. Recently, a study on HepG2 cells has demonstrated the intracellular localization of internalized-holo-HDL particles mainly in MVBs and only to a minor degree in lysosomes [14].

In the study reported here, we did not find any evidence for a resecretion of HDL by EPCs but we could clearly demonstrate a lysosomal intracellular pathway for HDL in these cells. Therefore, we suggest that HDL is capable to deliver cholesterol to the cells directly via binding of its degradation products.

At all periods of treatment, the number of stained organelles including MVBs, apparently increased with longer HDL incubation times. The highest number of positively stained organelles was found after 3 hours of incubation but decreased after 4 hours of incubation. This result corresponded to that achieved by fluorescence microscopic data of time course of the HDL-Alexa Fluor® 568 internalization. We suggest that with longer incubation periods, the HDL particles disappeared due to degradation corresponding to the increased presence of large vacuoles of secondary lysosomes after 3 hour of incubation. The lysosomes were positioned in close vicinity to the Golgi apparatus from which they were generated.

Along the endocytic pathway, many of HDL-positive compartments could be classified as MVBs, working as late endosomes. In EPCs, they could be demonstrated between 30 minute and 4 hours of incubation while unstained MVBs were also found. Positively stained MVBs often showed strong labeling of their vesicular content. In EPCs, the population of positive MVBs was not so frequently observed as in experiments on human HepG2 cells where the MVBs were the central HDL containing compartments [14]. However, these results are in agreement with our data underlining, that the reaction product positive MVBs increased in size and numbers, whereas negative MVBs decreased over the time course of the experiments. Our observation indicated that with longer incubation periods, the reaction product positive MVBs with irregular shape and tubular membranous extensions, increasingly appeared. These tubular extensions of MVBs might play a role in the delivery of recycling molecules.

It is interesting to note that the HDL-HRP containing endocytic compartments were arranged as aligned endosomes which appeared at 30 minutes up to 4 hours of incubation. In some cases, their membranes fused together and formed “strings of pearls-like structures” still having a single membrane. Two possible explanations can be proposed: 1) Since we found that HDL-positive endosomal vesicles increased in numbers over the time of the experiments, this arrangement might inhibit early endosome motility in the dynamics of the endosomal pathway in order to delay endosome maturation into later compartments. 2) This arrangement might be a precursor of Weibel Palade bodies that would be completely develop only in mature ECs.

The occurrence of numerous autophagosomes, frequently enclosed by doubled or multilamellar membranes containing engulfed endosomal vesicles and lysosomes is a striking feature of EPCs. The number of these structures increased with time of uptake, corresponding to the presence of the increasing number of stained organelles as well as lysosomes, presumably helping to maintain a balance between synthesis and degradation of cellular lipoprotein products. The intracellular traffic routes are summarized in a flow chart (Figure 13).

In conclusion, we were able by using cytochemical methods including fluorescence and electron microscopy to monitor the intracellular traffic routes of HDL along the endocytic pathway and concomitantly its degradation. We propose that the highly differential distribution of HDL along the endocytic pathway might be the result of efficient lipid sorting processes.
